# Flexor Pollicis Longus Weakness Post-proximal Radius Shaft Open Reduction and Internal Fixation: A Case Report

**DOI:** 10.7759/cureus.62423

**Published:** 2024-06-15

**Authors:** Sara G Qadi, Shahad Alshaynawi, Anmar Alkindy, Ghazi Aldalbahi

**Affiliations:** 1 Orthopedic Surgery, King Saud bin Abdulaziz University for Health Sciences, Jeddah, SAU; 2 Orthopedic Surgery, King Fahad General Hospital, Madinah, SAU; 3 Orthopedic Surgery, Ministry of National Guard Health Affairs, Jeddah, SAU; 4 Orthopedic Surgery, King Saud University, Riyadh, SAU

**Keywords:** fpl branch of ain, electrodiagnostic studies, radius shaft fracture, flexor pollicis longus, anterior interosseous nerve

## Abstract

We report the case of a patient who sustained a right proximal radial shaft fracture. He experienced isolated flexor pollicis longus weakness as a result of a partial anterior interosseous nerve (AIN) injury. The incidence of AIN injury is recognized as an exceptional postoperative complication for this particular type of fracture. It might be helpful to do electrodiagnostic investigations to confirm the diagnosis. A complete clinical recovery of the nerve occurred 16 weeks following the surgical operation.

## Introduction

Proximal radius fracture is one of the most common fractures in adults [1]. Usually, its management is open reduction internal fixation because it is difficult to reduce and maintain through healing in a cast [2]. However, the nerves and vessels located within or near the proximal radius, such as the posterior interosseous nerve (PIN) and anterior interosseous nerve (AIN), may get injured during the operation and complicate the surgery. Nevertheless, common risks during surgery result in PIN, recurrent radial artery, or superficial radial nerve injuries [3]. Moreover, AIN injury is considered a rare complication of post-proximal radius fixation with plates and screws. This report aims to describe the case of an adult patient who sustained an isolated right proximal radius shaft fracture and developed a partial AIN injury, resulting in isolated flexor pollicis longus (FPL) weakness confirmed by electromyography (EMG) post-fixation. In addition, it describes the expected recovery of a partial AIN injury isolated from the FPL.

## Case presentation

**Figure 1:**
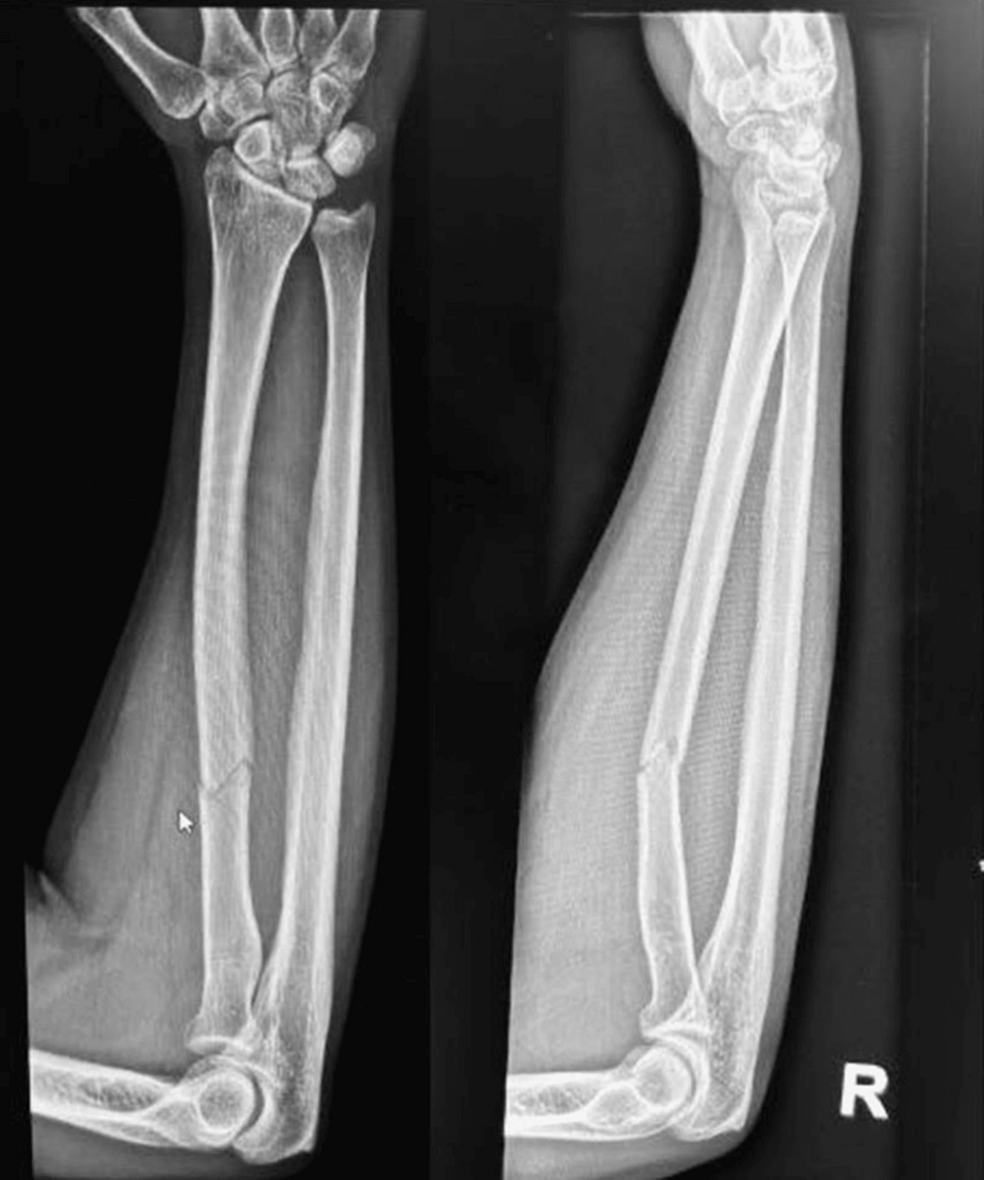
Preoperative AP and lateral view X-rays of the right forearm demonstrating proximal radial shaft fracture

We present the case of a 34-year-old medically fit male who is right-handed dominant. After falling on an outstretched hand (FOOSH), he was injured in a motor vehicle accident and arrived at our emergency room with right forearm pain and swelling upon presentation. Physical examination revealed a slight enlargement without an open wound, pain across the right proximal radial shaft, and full range of motion in the wrist and elbow, but limited pronation and supination. The neurovascular examination was intact, including flexion of the interphalangeal joint of the thumb. Plain radiographs revealed a right radius shaft fracture. Plain radiographs demonstrated a displaced fracture involving the right radius shaft (Figure 1).

The patient underwent open reduction internal fixation of the right proximal radial shaft. Through Henry’s approach, the incision was made lateral to the biceps tendon proximal to the mid-forearm, 8 cm in length, and then a subcutaneous incision was made up to the fascia. After retracting the flexor carpi radialis and brachioradialis muscles, a deep dissection of the supinator muscle was performed, taking PIN into careful consideration. We distally released some pronator teres fibers to position the plate on the bone. Two Hohmann retractors are placed in position to retract the muscles laterally and medially. Another two Hohmann retractors were added distally. The fracture was identified and cleaned. There was a butterfly that was partially broken and had plastic deformation anteriorly. The plate sat better on the posterolateral aspect. A DCP plate with six holes was utilized and held in place with a bone holder. First, two screws, one centric and one eccentric, were inserted. The proximal screws were 18 mm long, and the distal screw was 16 mm long. The fracture site was anatomically reduced under fluoroscopy. There were no intraoperative complications. Following the evaluation of fracture reduction, proper hemostasis was maintained while positioning the plate and screws. Although the original wound was closed, the fascia was left unclosed to reduce the possibility of edema. After closure, a backslap was applied above the elbow.

On the first postoperative day, while examining the patient, we found that the distal FPL failed to function, but the sensation was intact. PIN, radial, and the remaining muscles supplied by the AIN were intact. The patient was scheduled to undergo further investigation, a forearm MRI, a nerve conduction study (NCS), an EMG, and another day of observation. There were no signs of compartment syndrome on the acceptable postoperative X-ray. He began with physiotherapy and was discharged on the second postoperative day.

**Figure 2:**
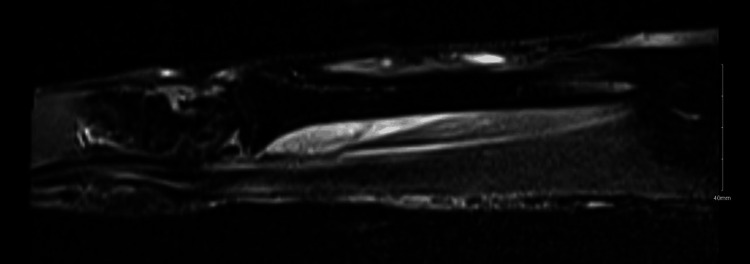
Distal FPL edema at the level of the distal radial diaphysis is evident on the MRI FPL, flexor pollicis longus

On a follow-up visit on postoperative day 14, the patient complained of the inability to flex the right thumb’s distal interphalangeal (DIP) joint. An MRI study was performed to exclude FPL tendon rupture. It showed edema of the distal FPL at the level of the distal radial diaphysis (Figure 2).

The finding could be related to denervation edema. At this time, the patient was kept on a backslap for another two weeks and planned to continue the physiotherapy program. However, electrodiagnostic studies were performed four weeks post-operatively. The NCS (Table 1) showed left median nerve to digit II mildly prolonged latency, normal amplitude, and mildly reduced conduction velocity. The presenting table showed normal sensory and distal motor latency, amplitude, and conduction velocity for muscles innervated by identifying nerves.

**Table 1:** NCS performed four weeks postoperative ADM, abductor digiti minimi; APB, abductor pollicis brevis; EDC, extensor digitorum communis; NCS, nerve conduction study; NCV, nerve conduction velocity

Motor NCS									
Site	Latency (ms)	Duration (ms)	Amplitude	Area	Stimulation (mA)	Segment	Distance (millimeter)	Interval (milliseconds)	NCV (m/s)*
Radial	Right radial nerve to the EDC muscle								
Wrist	3.7	9.6	3.7 mV	12.0 mVms	100	Wrist	80	3.7	N/A
Elbow	4.8	8.9	3.0 mV	11.0 mVms	100	Wrist-elbow	80	1.2	62.1
Median	Left median nerve to the APB muscle								
Wrist	3.3	4.9	10.8 mV	27.2 mVms	26	Wrist	70	3.3	N/A
Elbow	7.9	4.9	10.3 mV	26.8 mVms	26	Wrist-elbow	250	4.6	54.9
Median	Right median nerve to the APB muscle								
Wrist	2.9	4.5	13.6 mV	33.4 mVms	27	Wrist	70	2.9	N/A
Elbow	7.4	4.7	12.5 mV	33.0 mVms	34	Wrist-elbow	240	4.5	53.3
Ulnar	Left ulnar nerve to the ADM muscle								
Wrist	2.3	7.9	6.9 mV	29.9 mVms	25	Wrist	70	2.3	N/A
Elbow	7.1	7.1	6.6 mV	21.0 mVms	33	Wrist-elbow	260	4.8	54.2
Above elbow	8.9	6.7	6.4 mV	22.0 mVms	33	Elbow-above elbow	100	1.8	55.6
Ulnar	Right ulnar nerve to the ADM muscle								
Wrist	2.2	6.2	8.2 mV	28.3 mVms	27	Wrist	70	2.2	N/A
Below elbow	6.6	6.5	7.6 mV	27.4 mVms	35	Wrist-below elbow	250	4.4	57.5
Above elbow	8.5	6.3	7.4 mV	26.6 mVms	35	Below elbow-above elbow	110	1.9	57.9
Sensory NCS									
Site	Latency 1 (ms)	Latency 2 (ms)	Amplitude	Area	Stimulation (mA)	Segment	Distance (millimeter)	Interval (milliseconds)	NCV (m/s)*
Median	Left median nerve to digit II								
Wrist	3	3.6	18.8 uV	0.6 uVms	32	Wrist	130	3	42.9
Median	Right median nerve to digit II								
Wrist	2.3	3.2	31.8 uV	5.0 uVms	25	Wrist	130	2.3	55.6
Ulnar	Left ulnar nerve to digit V								
Wrist	1.6	2.4	24.0 uV	2.5 uVms	26	Wrist	110	1.6	67.9

EMG (Table 2) was performed, and there were neurophysiological features of denervation potential involving the right FPL. Right, FPL (close to the radial) showed 3+ spontaneous activity, large motor unit potential (MUP), a severely reduced recruitment pattern, and no neurophysiological features of reinnervation thus far. In addition, mild left median neuropathy across the wrist was present as an incidental finding. However, the patient was asymptomatic from that perspective. The results suggested a right partial AIN injury.

**Table 2:** EMG four weeks postoperative EMG, electromyography; FDP, flexor digitorum profundus; FPL, flexor pollicis longus; MUP, motor unit potential

EMG			
Muscle tested	Spontaneous activity	MUP	Recruitment
Right FDP 1	Normal	Normal	Normal
Right FDP 2	Normal	Normal	Normal
Right pronator quadratus	Normal	Normal	Normal
Right FPL	3	No MUP	No recruitment
Right FPL (close to radial)	3	Large MUP	Decreased

After nine weeks, an additional follow-up electrodiagnostic study revealed AIN neuropathy. The NCS (Table 3) showed normal sensory and distal motor latency, amplitude, and conduction velocity for the sites of tested muscles and nerves. There were diffuse denervation and innervation potentials, with some muscles demonstrating fasciculation potentials. Right flexor digitorum profundus (FDP 1), FDP 2, and right pronator quadratus showed no spontaneous activity, normal MUP, and normal recruitment. Right, FPL showed 3+ spontaneous activity, no MUP, and no recruitment (Table 4). The findings are likely due to AIN neuropathy. In conjunction with the prior study, there are overall clinical and neurophysiological improvements in patient function.

**Table 3:** Follow-up NCS performed nine weeks postoperative ADM, abductor digiti minimi; APB, abductor pollicis brevis; EDC, extensor digitorum communis; NCS, nerve conduction study; NCV, nerve conduction velocity

Motor NCS									
Site	Latency (ms)	Duration (ms)	Amplitude	Area	Stimulation (mA)	Segment	Distance (millimeter)	Interval (milliseconds)	NCV (m/s)*
Median	Right median nerve to the APB muscle								
Wrist	3.1	5.2	6.2 mV	17.8 mVms	39	Wrist	70	3.1	N/A
Elbow	7.6	5.2	5.3 mV	15.7 mVms	32	Wrist-elbow	250	4.5	55.6
Ulnar	Right ulnar nerve to the ADM muscle								
Wrist	2.8	5.2	6.3 mV	20.2 mVms	57	Wrist	70	2.8	N/A
Below elbow	7.2	5.6	5.4 mV	20.1 mVms	57	Wrist-below elbow	250	4.4	57.5
Above elbow	8.6	6.4	5.5 mV	19.9 mVms	57	Below elbow-above elbow	90	1.4	64.3
Radial	Right radial nerve to the EDC muscle								
Elbow	3.4	6.2	3.3 mV	8.6 mVms	100	Elbow	110	3.4	N/A
Above groove	5.1	6.1	2.8 mV	7.7 mVms	100	Elbow-above groove	100	1.7	58.8
Sensory NCS									
Site	Latency 1 (ms)	Latency 2 (ms)	Amplitude	Area	Stimulation (mA)	Segment	Distance (millimeter)	Interval (milliseconds)	NCV (m/s)*
Median	Right median nerve to digit II								
Wrist	2.6	3.2	32.4 uV	2.0 uVms	16	Wrist	130	2.6	50.4
Ulnar	Right ulnar nerve to digit V								
Wrist	2.1	2.6	26.2 uV	1.3 uVms	15	Wrist	110	2.1	52.4
Radial	Right radial nerve								
Forearm	1.7	2.2	35.3 uV	1.6 uVms	13	Forearm	100	1.7	57.5

**Table 4:** Follow-up EMG performed nine weeks postoperative EMG, electromyography; FDP, flexor digitorum profundus; FPL, flexor pollicis longus; MUP, motor unit potential

EMG			
Muscle tested	Spontaneous activity	MUP	Recruitment
Right FPL	3	No motor units	No recruitment
Right FDP 1	0	Normal	Normal
Right FDP 2	0	Normal	Normal
Right pronator quadratus	0	Normal	Normal

**Figure 3:**
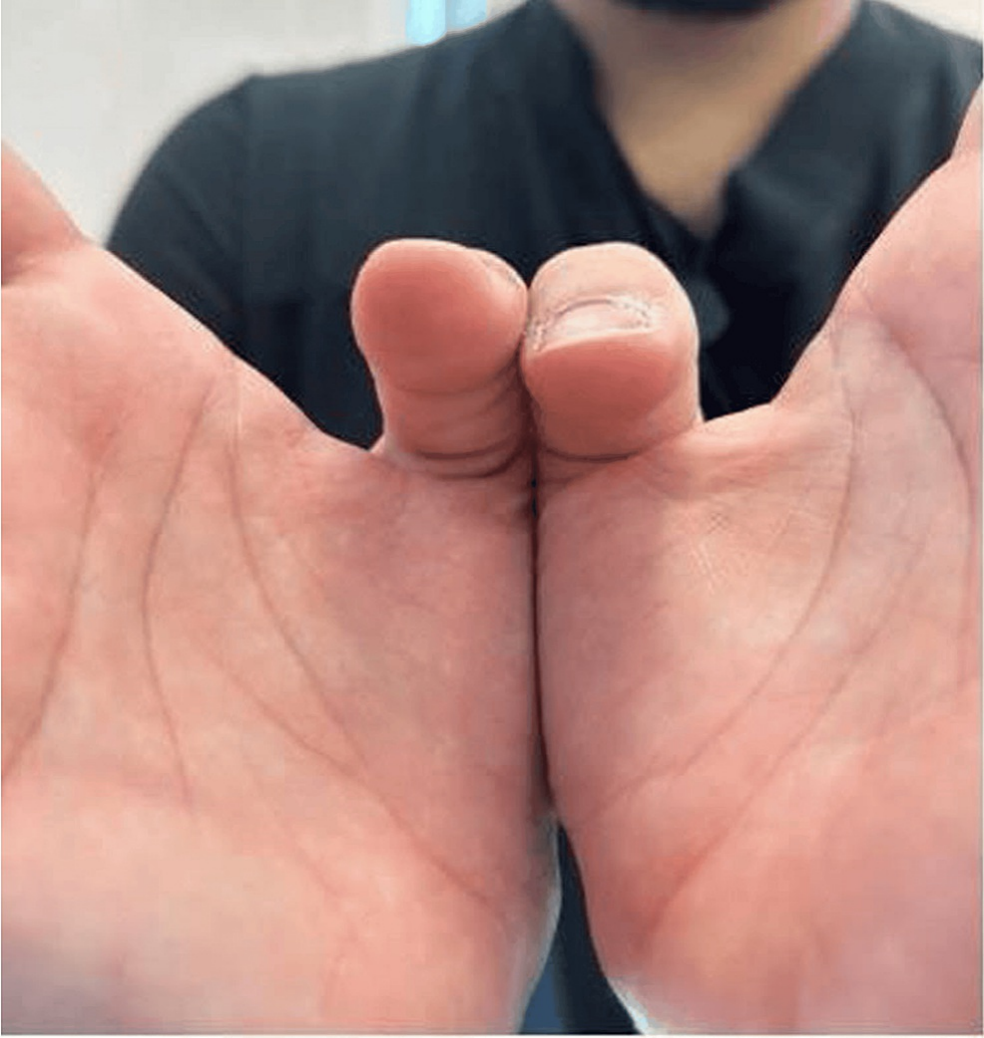
Seven weeks postoperative, showing improved right thumb DIP flexion DIP, distal interphalangeal

**Figure 4:**
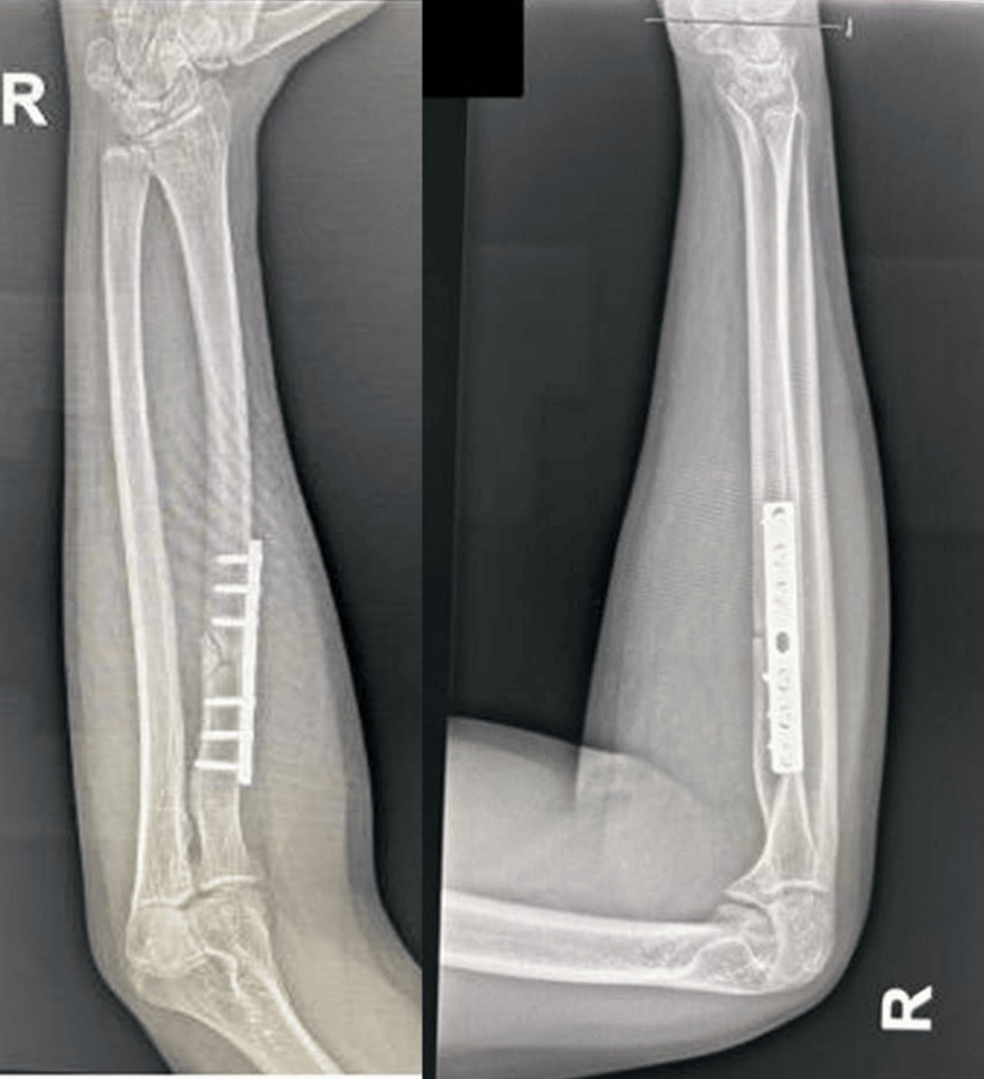
Postoperative AP and lateral view X-rays of the right forearm

**Figure 5:**
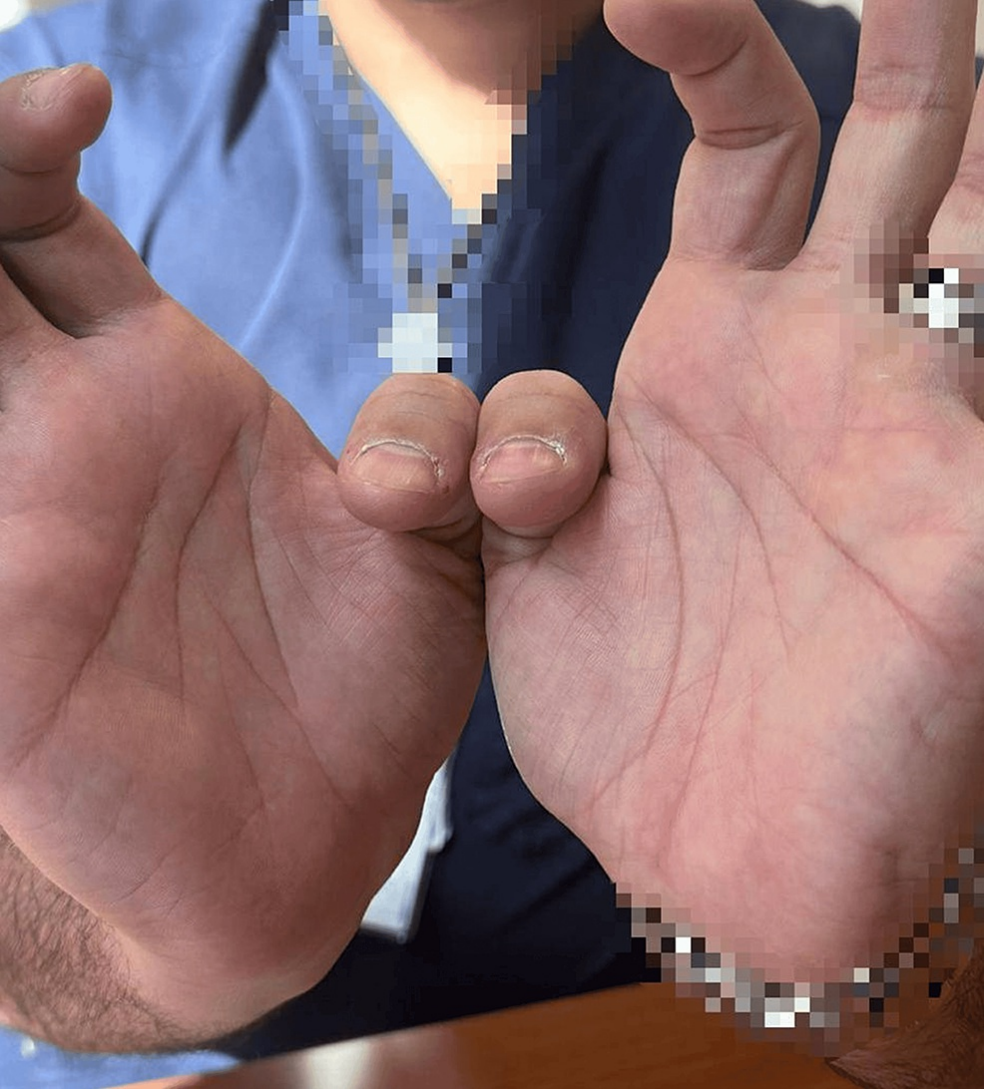
Sixteen weeks postoperative, showing complete recovery

At follow-up seven weeks postoperatively, the patient has improved in strength with active right thumb DIP flexion (Figure 3), and the postoperative X-ray showed acceptable healing (Figure 4). Full clinical recovery was achieved after 16 weeks postoperatively (Figure 5).

## Discussion

The AIN is the largest branch of the median nerve. It arises from its radial aspect, 5-8 cm distal to the level of the lateral condyle, and passes between the two heads of the pronator teres. It runs along the volar surface of the flexor digitorum profundus and then passes between the flexor digitorum profundus and the FPL on its way to the pronator quadratus. AIN causes flexion to the DIP joint of the thumb and index finger, which can be examined by an ok sign; in the case of isolated FPL, the patient will be unable to do flexion of the DIP joint of the thumb, but he will be able to do flexion to the DIP joint of the index finger.

Injury to the AIN is infrequent and accounts for 1% of upper limb neurological injuries [4]. Injury to the AIN nerve is not mentioned as a complication following the Henry approach, which utilizes the interval between the brachioradialis and flexor carpi radialis [5]. In our search, an isolated FPL injury following radial plating was found in two case reports in which both patients recovered fully. In the study done by Campbell et al., full recovery without complications was achieved after six months postoperatively [6]. In contrast, there is a study by Salkinder and Liebenberg. A patient presented with an open midshaft radius and an ulna fracture. Postoperatively, after open reduction and internal fixation of the fractured site, the patient experienced a loss of FPL function. He achieved full FPL recovery after 10 weeks postoperatively [7]. After the operation, the patient had weakness on the first day with preserved sensation. Then, seven weeks after the operation, he started to see an improvement in the FPL function. The nerve achieved complete clinical recovery 16 weeks after the operation.

The cause of postoperative FPL injury could be due to several factors, including anatomical variations as described by Dolderer et al.’s study [8], or could be related to other causes as described in other studies, such as hematoma, edema, or extensive stripping of the FPL muscle [9]. After the surgery, an MRI was performed, which revealed swelling in the distal FPL near the distal radial diaphysis. It is possible that this swelling is related to the initial trauma or excessive traction during the surgery. In all studies, there was complete recovery of FPL muscle function, but we need to ensure this injury is not related to muscle rupture by doing an MRI and NCS.

## Conclusions

The sustained weakness of FPL due to an AIN injury after radius plating is considered a rare phenomenon. Several factors could contribute to this injury, such as traction neuropraxia of the motor branch to the FPL, anatomical variations, hematoma, edema, or extensive stripping of the FPL muscle. If a loss of FPL function is detected after surgery, it is recommended to initially take a conservative approach.
